# Exploiting deep learning and volunteered geographic information for mapping buildings in Kano, Nigeria

**DOI:** 10.1038/sdata.2018.217

**Published:** 2018-10-23

**Authors:** Jiangye Yuan, Pranab K. Roy Chowdhury, Jacob McKee, Hsiuhan Lexie Yang, Jeanette Weaver, Budhendra Bhaduri

**Affiliations:** 1 Urban Dynamics Institute, Oak Ridge National Laboratory, Oak Ridge, TN, USA; 2 University of Tennessee, Knoxville, TN, USA

**Keywords:** Geography, Developing world, Databases

## Abstract

Buildings in the developing world are inadequately mapped. Lack of such critical geospatial data adds unnecessary challenges to locating and reaching a large segment of the world’s most vulnerable population, impeding sustainability goals ranging from disaster relief to poverty reduction. Use of volunteered geographic information (VGI) has emerged as a widely accepted source to fill such voids. Despite its promise, availability of building maps for developing countries significantly lags behind demand. We present a new approach, coupling deep convolutional neural networks (CNNs) with VGI for automating building map generation from high-resolution satellite images for Kano state, Nigeria. Specifically, we trained a CNN with VGI building outlines of limited quality and quantity and generated building maps for a 50,000 km^2^ area. Resulting maps are in strong agreement with existing settlement maps and require a fraction of the manual input needed for the latter. The VGI-based maps will provide support across multiple facets of socioeconomic development in Kano state, and demonstrates potential advancements in current mapping capabilities in resource constrained countries.

## Background & Summary

Individual building locations and extents are the foundation of maps that support a broad range of applications requiring spatial awareness of population and associated infrastructures. Using active remote sensing data such as LiDAR to build maps is the most common practice. However, due to higher costs of this technology, these data are severely lacking for developing nations, presenting major challenges for critical tasks such as locating vulnerable populations during humanitarian crises (e.g., disease epidemics, conflict, natural disasters) and improving policy and decision making to achieve long-term development goals. Due to the increasing availability of very-high-resolution (<1 m) satellite images, creating and routinely updating accurate building maps reveal encouraging promise. However, manually mapping buildings from images is notoriously cost- and time-intensive. Volunteered geographic information (VGI)^1^ provides an important solution by crowdsourcing large-scale mapping efforts. VGI-based mapping capabilities are bounded, however, by a locality’s level of community engagement and thus remains largely insufficient in terms of consistency and sustainability. OpenStreetMap (OSM), which was launched in 2004 and represents one of the most successful VGI-based mapping projects, has mapped only a fraction of buildings in developing countries. For example, the amount of buildings mapped for the entire continent of Africa is equivalent to those mapped in Germany, just one country (this comparison is based on map data size). More importantly, given the voluntary nature of mapping with VGI, it is difficult to ensure data quality and fidelity, which has been found to vary significantly across different geographic regions^2,3^.

Kano state, Nigeria, was selected as the study site because the area exhibits widely varying geographic features. It is situated in the Sahel, a transitional region between the Sahara to the north and the Sudanian Savanna to the south. Due also to the diversity of ethnic groups inhabiting the region and varied ecological conditions^4^, both the social and natural environments have aided in the creation of highly distinct building types and development patterns across Kano state.

In this paper, we present a highly complete building map for Kano state, Nigeria, generated by combining deep learning techniques with VGI data. The map is expected to provide a key data layer for a wide range of socioeconomic applications, which could further validate our mapping approach. Compared to pure VGI approaches, this approach greatly reduces reliance on participation levels and addresses concerns regarding uncertain data quality. Our method also leverages state-of-the-art machine learning capabilities without requiring massive data annotation efforts. Low resource usage and high efficiency make our approach particularly appealing for applications in developing countries, where it can be difficult to use existing methods.

## Methods

We introduce a novel approach that utilizes deep learning methods coupled with minimally required open source, publicly available, VGI data for large-scale mapping of buildings. Specifically, we use OSM building outlines to generate labeled images for training convolutional neural networks (CNNs), developing new strategies to overcome limitations inherent to VGI-derived labeled data and enabling networks to generalize to areas thousands of times larger than those covered by existing building maps.

Since deep CNNs trained with large volumes of labeled data have displayed unprecedented levels of performance in extracting information from visual data^5–9^, numerous studies have explored similar techniques for mapping buildings from remotely sensed images^10–12^. However, these methods use labeled data generated from carefully controlled efforts, which are expensive to scale up, and results are only validated on small geographic regions.

### Data

**Figure 1:**
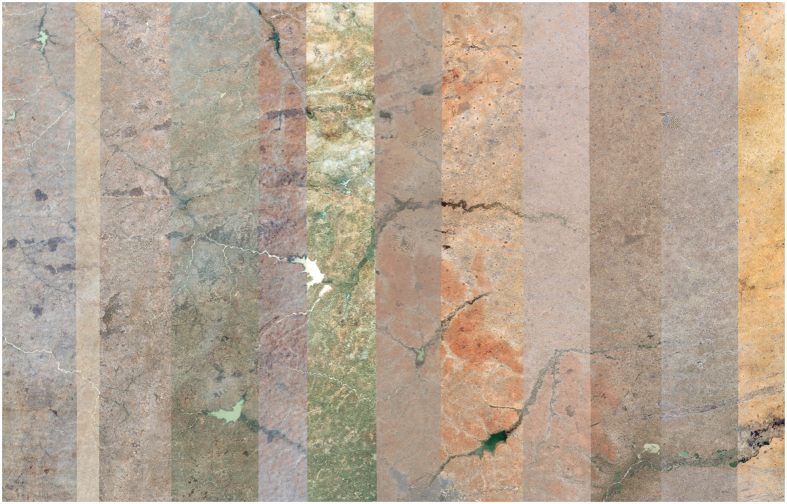
Example satellite images of northern Kano state.

Our image collection contains 30 natural color (RGB), partially overlapping image strips of size 270,000×35,000 pixels (~ 0.5 m resolution) from WorldView-2 satellite and covers an area of 50,000 km^2^. Satellite images were acquired between February 2010 and November 2013. We did not impose any special requirements on acquisition characteristics, such as viewing angles or solar angles, when selecting images. Figure 1 shows image strips covering northern Kano state. Physiographic features within such a vast area dramatically vary. Because images are collected at different times, significant variations can be observed across image strips due to disparate lighting conditions brought about by seasonality, sun angle, and/or sun elevation.

Within the study region, OSM has 80,000 building polygons. Although the actual number of buildings is unavailable, the following comparison provides some quantitative insights into the scarcity of building data. Washington, DC, which has approximately 160,000 buildings, is only one-eighteenth as populated and 100 times smaller than Kano state. Within this area, only the capital city, Kano, has relatively complete building maps. Southern Kano state is more vegetated, exhibiting geographic features quite different from those in the city of Kano. To develop a more comprehensive labeled training dataset, we also collected building map data from the city of Yaounde, Cameroon, in the Sudanian Savanna. OSM building layers are overlaid with corresponding images to yield a labeled dataset, where a simple procedure is applied to reduce misalignment between maps and images^13^. The resulting dataset includes 820 images of size 500×500 pixels (network input dimension), which represents 0.05% of the size of all images to be processed.

### CNN-Based Method

We utilize a CNN architecture designed for spatial object extraction^14^. The network is capable of pixel-level prediction and orders of magnitude smaller in size than other networks used for natural image analysis^15,16^, providing for faster processing speed. The network has been shown to produce highly accurate building extraction results on a city-scale dataset.

One major challenge in our task is the insufficient quantity of labeled data. The area covered by previously mapped data is extremely small compared to the area that still needs to be mapped. It is widely recognized that CNNs struggle to perform adequately with small labeled datasets. To address this problem, we utilized a strategy of multiple rounds of training with limited human feedback. We started with training only from OSM data. The trained network was tested on a set of randomly selected areas. Image analysts were assigned to identify errors in the results and make corrections. This procedure was intended to collect a small amount of additional labeled data. The network is then initialized with the previous model and retrained with the expanded training data. The train-and-feedback cycle can be repeated as needed. Our experiment showed such feedback information can effectively improve generalization, and just two rounds of training resulted in a network that works sufficiently well over broad regions.

The network has seven regular ConvNet stages, each of which consists of a convolutional layer and an optional max-pooling layer. Convolutional layers of the 7 stages have 50 filters of size 5×5×3, 70 filters of size 5×5×50, 100 filters of size 3×3×70, 150 filters of size 3×3×100, 100 filters of size of size 3×3×150, 70 filters of size of size 3×3×100, and 70 filters of size of size 3×3×70, respectively. In the first four stages, the convolutional layer is followed by a max-pooling layer over a 2×2 unit region. The final stage takes the output feature maps of the first, second, third, and seventh stages, where feature maps are stacked together via upsampling. A 1×1×*n* filter (*n* being the depth of the feature map stack) is then applied to each pixel location to produce a prediction. Due to the use of feature maps at different stages, such a prediction exploits information at multiple scales. The signed distance function of boundaries has been shown to be a well-suited output representation, where the value at each pixel is its distance to the nearest boundary pixel with signs indicating inside and outside boundaries. The final stage uses 128 filters, each of which outputs the probability of a pixel falling in one of the equally divided distance bins between –128 and 128. All layers apply rectified linear unit nonlinearity. This network takes three-band input images that can be of arbitrary size. The network output can be considered a class distribution with each distance bin representing a class. During training, the cost function is defined as the cross entropy between the output after softmax normalization and the labeled data in signed distance form.

The network was trained with stochastic gradient descent with a mini-batch size of 5. We employed a widely used weight update rule^4^ with a learning rate of 0.01. The only preprocessing on images involved computing the mean of each band of all training images and subtracting them from each pixel. The first round of training used the labeled dataset from OSM and took 25 h.

We applied the trained network to processing new images. As in training, the only preprocessing involved mean subtraction. At each pixel location, softmax-normalized output of each filter at the last stage indicates the probability of the pixel falling into the corresponding distance bin, and the expectation is taken as the final output. Positive pixels are marked as buildings. While the network produces satisfying results for large areas, it has difficulty dealing with scenarios that are significantly different from those in the training set. For example, in rural areas, many isolated settlements are missed, and patterns that happen to have certain regular geometric features are falsely detected.

We generated results for randomly selected images and assigned four image analysts to visually inspect the results and provide correct annotations. In two hours, we collected 60 images (500×500 pixels) with buildings delineated and 100 images containing only negative samples, where entire images are labeled as negative without any delineation. The network was initialized with the parameter values from the first round of training and retrained using the training set with new labeled data.

**Figure 2:**
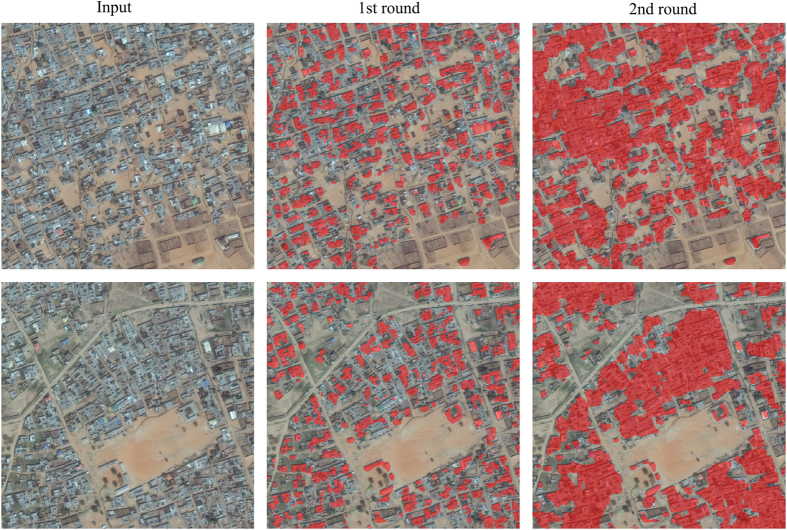
Building map improvement following two rounds of training. The network learns to group dense buildings when trained with small amounts of additional data that are similarly labeled. Extracted buildings are marked in transparent red.

The network used the additional training data effectively, and results were markedly improved. The network now identified many rural buildings that were previously missed and correctly rejected patterns not related to buildings. In the first round, our efforts were challenged by densely situated buildings, making individual building difficult to identify (Fig. 2), and by the lack of meaningful labels in OSM data. To minimize annotation efforts and maximize useful results, analysts were instructed to draw boundaries of neighborhoods containing buildings that are connected. As can be seen in Fig. 2, the retained network groups dense buildings together while at the same time precisely delineating individual buildings. Note that these improvements result from a small amount of manual editing. This trained network was used to process the whole image set, which took two days.

To highlight the baseline efficiency of our method, all experiments are conducted using a single NVIDIA Tesla K80 GPU. With more GPUs available, training time can be reduced by implementing parallel training methods^17,18^. When applying the training network for production, processing speed increases linearly with the number of GPUs by simply distributing data to multiple units. For example, we processed the same image set using two GPUs, which took only one day.

### Code Availability

Source code is available upon request.

## Data Records

Original mapping results are raster data with the same resolution as input satellite images (0.5 m). To facilitate geospatial analysis, output raster images were converted into vector data and combined into a single feature class, a standard geospatial data format. The data are accessible for download at (see 1) and https://udi.ornl.gov/content/data.

## Technical Validation

**Figure 3:**
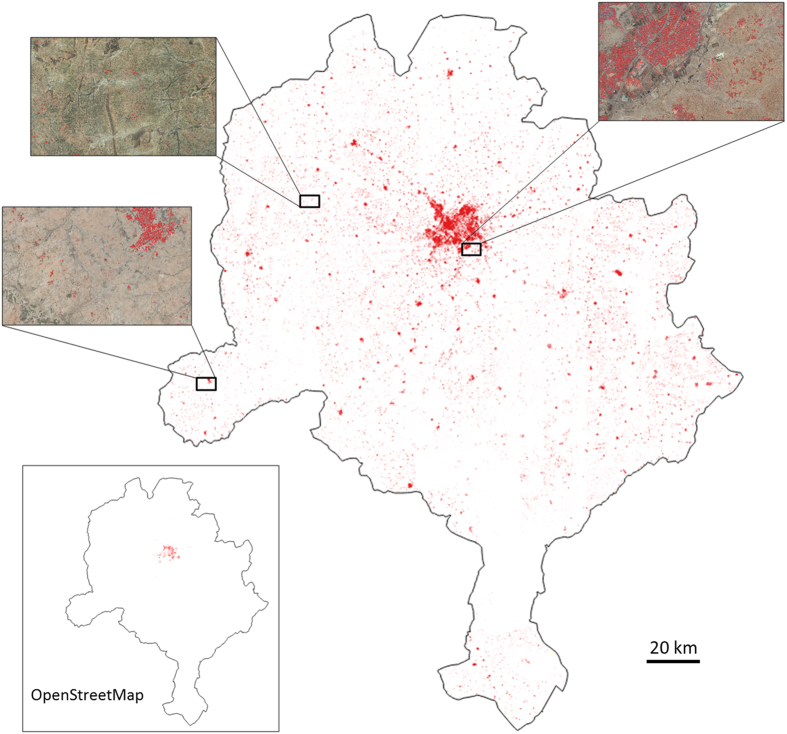
Building map generated for Kano state. All extracted buildings are marked in red. Three zoom levels are provided, with buildings overlaid on images. OSM building footprint data, predominantly located in Kano city, are visualized in the same manner.

**Figure 4:**
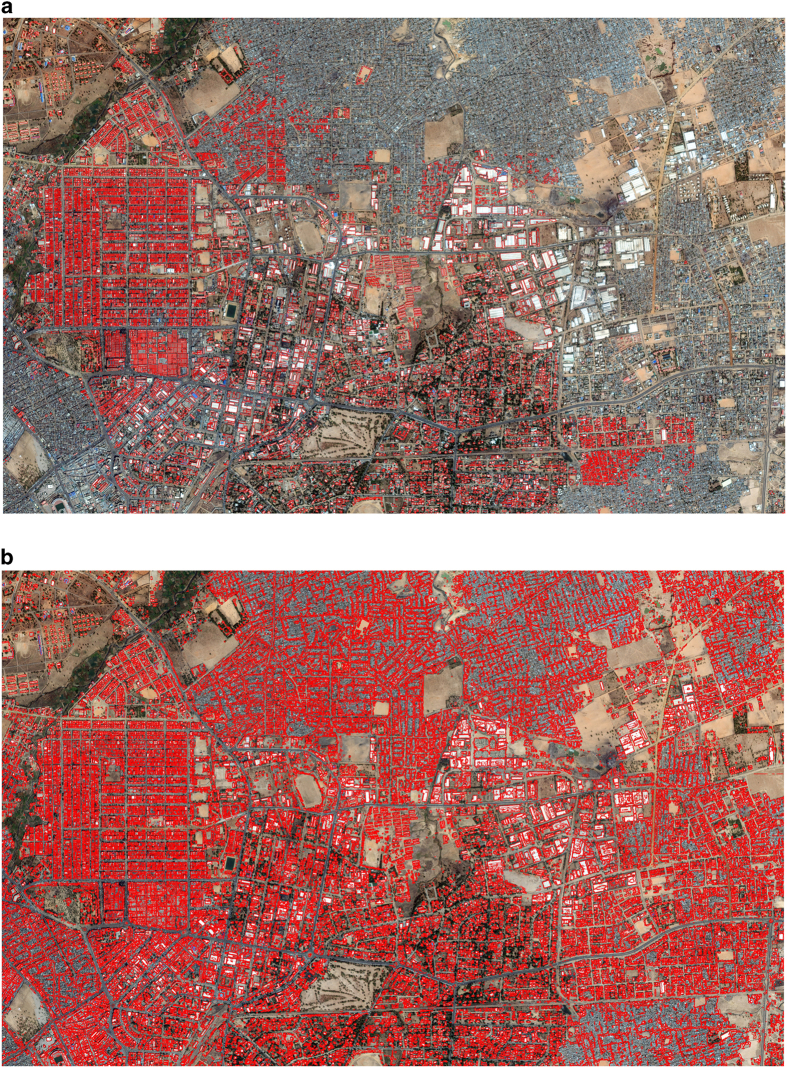
Comparison of map completeness for Kano city. (**a**) Building map generated using OSM data. (**b**) Building map generated using our approach. Building boundaries are marked in red.

The resulting building map for Kano state is illustrated in Fig. 3, which shows that the network generates a comprehensive identification and representation of buildings. To the best of our knowledge, most buildings in this region did not appear on any digital map prior to this experiment. The inset shows the building footprint data from OSM, which are virtually non-existent outside Kano city. For places with available OSM building footprints, our work also results in a more complete map (Fig. 4).

Large-scale quantitative evaluation of the results is challenging due to the lack of reference data. Assuming the same productivity rate achieved while generating the additional labeled data as an approximation for manual delineation of buildings, 56,000 man-hours would be required to delineate buildings for the entire image set. To make the problem more tractable, we compared the results against existing settlement maps. In remote sensing efforts, settlements are defined as units detected by certain sensors as containing buildings^19^. Although settlement maps often have coarse resolution and do not aim to delineate individual buildings, they indicate the presence of buildings and thus can be used to assess the accuracy of our building extraction results at the corresponding resolution. We use settlement layers developed at Oak Ridge National Laboratory^20^ that were generated using the same set of imagery. The settlement map for Kano state was created using extensive quality control procedures and has a spatial resolution of 8 m, the highest resolution data available for this area to our best knowledge. The settlement map was produced using an automated tool combined with manual refinement. The tool computes low-level image features and produces initial classification, which is then verified, validated, and modified by image analysts. The settlement map for the target area is produced using the same collection of images and requires approximately 480 man-hours to complete.

For evaluation, we map building extraction results to the same grid defined by settlement maps, where cells containing any building pixels are positive and others are negative. For binary classification, the precision–recall metric is commonly used to quantitatively evaluate results. Precision is the fraction of detected building pixels that matches settlement maps, while recall is the fraction of positive pixels in settlement maps that is identified in building maps. The harmonic mean of two quantities, referred to as the F-score, provides a unified measurement. For the entire Kano state, the building extraction results achieved a precision rate of 0.721 and a recall rate of 0.703. Worth noting is that many discrepancies between two maps are attributed to the fact that settlement maps identify areas related to any human habitation or settlements by generalizing building perimeters and including space within close proximity of buildings, while building extraction results are more exclusive to buildings themselves.

To put the evaluation scores in perspective, we also evaluated two other automatically generated settlement maps available in this area: Global Human Settlement Layer (GHSL)^19^ created by the Joint Research Center at the European Commission and Global Urban Footprint (GUF) available from the German Aerospace Center^21^. GHSL was created using Landsat imagery, for which classification is based on multiscale texture and morphological image features extraction. The data layer has a spatial resolution of 38 m. Input data for GUF included TerraSAR-X and TanDEM-X amplitude images, both of which are radar images measuring surface elevation. Texture features were extracted from images to classify areas settled or non-settled. GUF has 12 m resolution. Two settlement maps were converted to the same resolution as the LandScan settlement map for precision and recall calculation. All scores are provided in Table 1. GHSL has rather low scores, having missed most small settlements and having included many roads. With height information available, GUF is much more accurate than GHSL, and its evaluation scores are only slightly lower than those of our building extraction result. However, it should be noted that at a similar resolution, a TerraSAR-X image is ten times as expensive as an optical image (price information can be found at http://www.intelligence-airbusds.com).

## Additional information

**How to cite this article**: Yuan, J. *et al*. Exploiting deep learning and volunteered geographic information for mapping buildings in Kano, Nigeria. *Sci. Data*. 5:180217 doi: 10.1038/sdata.2018.217 (2018).

**Publisher’s note**: Springer Nature remains neutral with regard to jurisdictional claims in published maps and institutional affiliations.

## Supplementary Material



## Figures and Tables

**Table 1:** Accuracy comparison for our method against other published settlement maps.

	Precision	Recall	F-score
GHSL	0.576	0.524	0.549
GUF	0.718	0.695	0.706
Building extraction	0.721	0.703	0.717
